# Changes in Antibiotic Resistance Patterns in Diabetic Foot Infections Requiring Toe Amputation: A Long-Term Single-Center Retrospective Study

**DOI:** 10.3390/antibiotics15070681

**Published:** 2026-07-10

**Authors:** Alaaddin Levent Özgözen, Enes Altunay

**Affiliations:** 1 Department of Orthopedics and Traumatology, Baskent University, Adana Dr. Turgut Noyan Training and Research Center, Adana 01250, Turkey; 2 Department of Medical Microbiology, Baskent University, Adana Dr. Turgut Noyan Training and Research Center, Adana 01250, Turkey; enes_altunay@hotmail.com

**Keywords:** diabetic foot, drug resistance, bacterial, anti-bacterial agents, amputation

## Abstract

**Objectives:** Diabetic foot infections are a major cause of morbidity and amputation, and increasing antibiotic resistance complicates their management. This study aimed to evaluate longitudinal changes in antibiotic resistance among bacterial pathogens isolated from patients undergoing toe amputation due to diabetic foot infections. **Methods:** This retrospective, single-center study included patients who underwent toe amputation for diabetic foot infections between 2013 and 2024. Microbiological culture results and antimicrobial susceptibility data were analyzed across three consecutive time periods: 2013–2016, 2017–2020, and 2021–2024. **Results:** A total of 351 patients were included (mean age, 64.1 years; 64% male). A history of dialysis was present in 30% of patients, and 54% had a history of prior hospitalization. A total of 351 patients were included in the study. A total of 378 bacterial isolates recovered from positive microbiological cultures were included in the antimicrobial susceptibility analysis. The most frequently isolated microorganisms were *Escherichia coli*, *Enterococcus* spp., coagulase-negative *Staphylococcus*, and *Staphylococcus aureus*. Among Enterobacterales isolates, statistically significant increases in resistance across the three consecutive time periods were observed for amoxicillin–clavulanate (*p* = 0.009), piperacillin–tazobactam (*p* = 0.002), trimethoprim–sulfamethoxazole (*p* = 0.012), and meropenem (*p* = 0.027), whereas the increase in imipenem resistance did not reach statistical significance (*p* = 0.054). Within *Staphylococcus* spp., a statistically significant increase in resistance across the three consecutive time periods was observed only for ciprofloxacin (*p* = 0.047). **Conclusions:** Changes in antimicrobial resistance rates were observed among bacterial isolates recovered from diabetic foot infections across the three consecutive time periods, highlighting the importance of regional surveillance and up-to-date local resistance data for guiding empirical antibiotic therapy.

## 1. Introduction

Diabetes is a major global public health concern, with a steadily increasing prevalence worldwide. According to recent estimates, approximately 589 million adults aged 20–79 years were living with diabetes in 2024, corresponding to 11.1% of the global adult population, and this number is projected to increase to 853 million by 2050 [1]. In Türkiye, analysis of the Turkish National Electronic Health Record (e-Nabız) system, encompassing approximately 64 million citizens and more than 99% of the adult population by the end of 2020, demonstrated a diabetes prevalence of 11.1%, with higher rates among women (13.1%) than men (9.1%) [2].

Diabetic foot complications are associated with substantial morbidity and mortality. Local data have shown that although microbiological cultures are obtained from only 60.5% of patients undergoing below-knee amputation due to diabetic foot complications, 73.9% receive empirical antibiotic therapy [3]. Furthermore, the prognosis of these patients is poor, with the mean post-amputation survival following below-knee amputation reported to be less than three years, a figure lower than that observed for many malignancies [4]. Beyond their clinical consequences, diabetic foot infection markedly impairs social and occupational functioning, with productivity losses estimated to exceed one-third of anticipated working time and job losses reported in up to 31.7% of affected individuals [5]. In addition to their functional and socioeconomic burden, the management of diabetic foot infections is increasingly challenged by antimicrobial resistance, which has been associated with inappropriate initial antimicrobial therapy, higher rates of reinfection, and poorer clinical outcomes [6]. These complications also impose a considerable economic burden, with diabetes-related healthcare expenditures in the United States estimated at approximately 237 billion USD annually [7].

Diabetic foot infections may be monomicrobial or polymicrobial and can involve both Gram-positive and Gram-negative organisms. The most commonly isolated pathogens include *Staphylococcus aureus*, *Pseudomonas* spp., *Escherichia coli*, and *Enterococcus* spp. [8]. However, the distribution of causative agents varies across regions and is influenced by geography. Recent evidence indicates that climatic and geographic factors contribute to regional differences in diabetic foot infection microbiology. A recent meta-analysis demonstrated that Gram-negative pathogens occur at significantly higher proportions in tropical and subtropical regions than in temperate climates [9]. Furthermore, not only pathogen distribution but also antimicrobial susceptibility profiles vary considerably across geographic regions [10].

Toe amputations are a commonly performed surgical procedure in patients with diabetic foot complications and are classified as minor amputations. In a large longitudinal cohort of 1729 patients with diabetic foot infection, toe amputations were required in 36.4% of cases, whereas major amputations were performed in only 6.5% of patients [7]. The need for toe amputation typically arises from advanced local disease characterized by deep soft-tissue infection, tissue necrosis, osteomyelitis, or localized gangrene. According to the Wagner classification system, these lesions most commonly correspond to grades 2–4, reflecting deep ulceration, bone involvement, and varying degrees of tissue loss [11].

Hemoglobin A1c (HbA1c) is a biomarker that reflects glycemic control over the preceding 8–12 weeks and is one of the most widely used indicators in diabetes management. Elevated HbA1c levels are associated with poor glycemic control and have been linked to impaired wound healing, increased susceptibility to infection, and poorer clinical outcomes in patients with diabetic foot infection. Therefore, assessment of glycemic control plays an important role in predicting prognosis and guiding treatment strategies in patients with diabetic foot disease [12].

Antibiotic consumption in Türkiye is higher than in many other countries [13]. Increased antibiotic use promotes selective pressure on microorganisms and facilitates the emergence of resistant strains. Patients with diabetic foot infections are particularly vulnerable to this process because they frequently require repeated courses of antimicrobial treatment, undergo multiple surgical procedures, and often suffer from chronic or recurrent infections. As a result, multidrug-resistant pathogens are commonly encountered in this patient population.

Therefore, this study aimed to evaluate the bacterial diversity in patients undergoing toe amputation for diabetic foot infections at our hospital between 2013 and 2024 and to assess changes in antibiotic resistance patterns across three consecutive time periods.

## 2. Results

This study collected data from 351 patients who underwent toe amputation due to diabetic foot infection between 2013 and 2024. Among these patients, 126 were female and 225 (64%) were male. The average age was 64.1 years. One hundred five patients (30%) were undergoing dialysis treatment for renal failure. Furthermore, 190 individuals (54%) had a history of previous hospitalization. The average HbA1c level at the time of amputation was documented as 8.9 ± 2% (Table 1).

**Figure 1:**
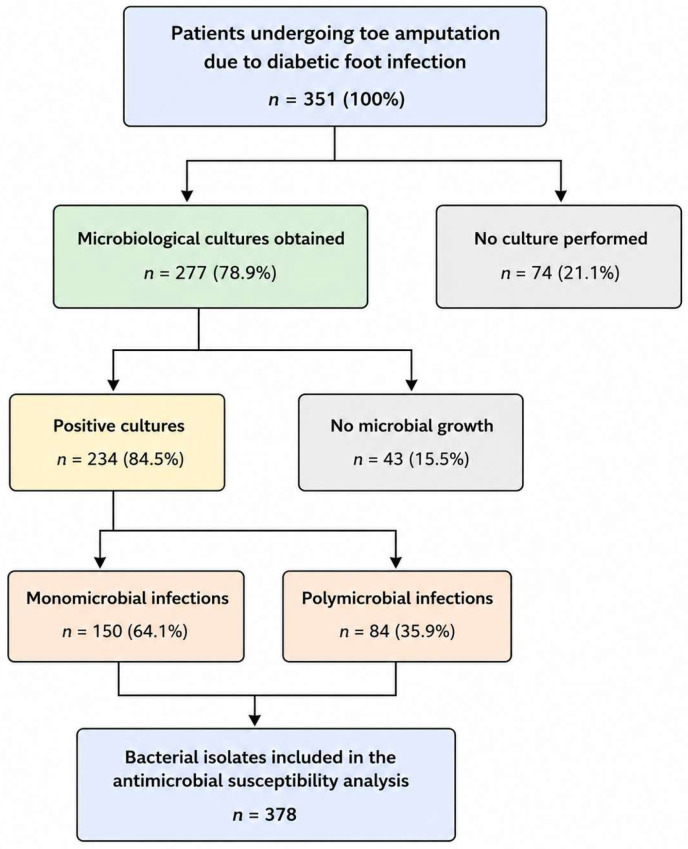
Flow diagram of patient inclusion and microbiological culture outcomes.

Microbiological cultures were obtained from 277 of the 351 patients (78.9%). Microbial growth was detected in 234 cultures (84.5%), whereas no growth was observed in 43 cultures (15.5%). Among the culture-positive specimens, 150 (64.1%) were monomicrobial and 84 (35.9%) were polymicrobial. A total of 378 bacterial isolates recovered from the positive cultures were included in the antimicrobial susceptibility analysis (Figure 1). A total of 378 microbial isolates representing 32 different microbial species (31 bacterial species and *Candida albicans*) were identified. Their distribution and classification into major microbiological groups are summarized in Table 2.

The four most frequently isolated microorganisms were *Escherichia coli* (14.8%), *Enterococcus* spp. (13.5%), coagulase-negative *Staphylococcus* (13.2%), and *Staphylococcus aureus* (9%).

When the bacterial profiles isolated from the patients were evaluated in relation to variables such as gender (female/male), history of dialysis, and prior hospital admission, no statistically significant differences were observed between the groups (Table 3).

A total of 167 Enterobacterales isolates were analyzed. No significant differences were observed in the proportions of patients undergoing dialysis or with a history of previous hospitalization across the three consecutive time periods (*p* = 0.686 and *p* = 0.673, respectively). Statistically significant increases in resistance were observed for amoxicillin–clavulanate (OR = 1.677; 95% CI: 1.137–2.474; *p* = 0.009), piperacillin–tazobactam (OR = 2.399; 95% CI: 1.389–4.146; *p* = 0.002), trimethoprim–sulfamethoxazole (OR = 1.701; 95% CI: 1.122–2.579; *p* = 0.012), and meropenem (OR = 2.779; 95% CI: 1.124–6.872; *p* = 0.027). Table 4 summarizes the antibiotic resistance rates of Enterobacterales isolates across the three consecutive time periods.

Although an increasing trend in imipenem resistance was observed across the three consecutive time periods, this increase was only of borderline statistical significance (OR = 2.463; 95% CI: 0.986–6.152; *p* = 0.054). Variations in resistance rates were also observed for the other antibiotics across the three consecutive study periods; however, none of these differences reached statistical significance (*p* > 0.05).

A total of 84 staphylococcal isolates were analyzed. No significant differences were observed in the proportions of patients undergoing dialysis or with a history of previous hospitalization across the three consecutive time periods (*p* = 0.128 and *p* = 0.330, respectively). A statistically significant increase in ciprofloxacin resistance was identified across the three consecutive time periods (OR = 1.822; 95% CI: 1.009–3.292; *p* = 0.047). Increasing trends in methicillin (*p* = 0.084), levofloxacin (*p* = 0.085), and fusidic acid (*p* = 0.132) resistance were also observed; however, these changes did not reach statistical significance. No resistance to vancomycin or teicoplanin was detected in any of the three consecutive time periods. Variations in resistance rates for the remaining antibiotics across the three consecutive time periods were not statistically significant (*p* > 0.05). Table 5 summarizes the antibiotic resistance rates of staphylococcal isolates across the three consecutive time periods.

Due to the limited numbers of *Enterococcus* spp. (*n* = 51), *Streptococcus* spp. (*n* = 25), and non-fermentative Gram-negative bacilli (*n* = 39) isolates, changes in antibiotic resistance across the three consecutive time periods were not analyzed for these groups. The overall antibiotic resistance rates for these organisms are summarized in Table 6.

## 3. Discussion

In this study, we evaluated the distribution of microorganisms and compared antimicrobial resistance patterns among bacterial isolates recovered from patients who underwent toe amputation for diabetic foot infection across three consecutive time periods. We observed statistically significant increases in resistance to amoxicillin–clavulanate, piperacillin–tazobactam, trimethoprim–sulfamethoxazole, and meropenem among Enterobacterales, as well as to ciprofloxacin among *Staphylococcus* spp. These findings demonstrate changes in institutional antimicrobial resistance patterns across the three consecutive time periods and underscore the importance of continuous regional microbiological surveillance to guide empirical antibiotic therapy and antimicrobial stewardship strategies.

Type 2 diabetes is typically diagnosed at around 52 years of age [15]. Within twenty years post-diagnosis, a significant percentage of individuals manifest peripheral artery disease and diabetic neuropathy [16,17]. The conjunction of diminished protective sensibility and advancing peripheral vascular disease significantly elevates the likelihood of foot ulcer development. Prior research indicates that diabetic foot infection predominantly arises in individuals aged 50 to 70 years and are more frequently detected in male patients [18,19]. This study involved toe amputations conducted at a mean age of 64.1 ± 10.7 years, with males representing the bulk of the cohort (64%), in accordance with existing literature.

Approximately half of end-stage renal disease cases are attributable to diabetes [20]. Hemodialysis patients are particularly prone to catheter-related infections, often requiring repeated antibiotic exposure [21]. In our cohort, nearly 30% of patients were undergoing hemodialysis, and 59% had underlying chronic kidney disease. The distribution of dialysis-dependent patients did not differ significantly across time-based groups. Among patients receiving dialysis, Enterobacterales accounted for the highest proportion of infections (52.8%); however, bacterial distributions were comparable between groups.

Hospitalization and inpatient antibiotic exposure are well-recognized risk factors for antimicrobial resistance [22]. In this study, 54% of patients had a history of prior hospitalization. No statistically significant differences were observed in hospitalization rates across time-based groups. The balanced distribution of dialysis-dependent and previously hospitalized patients across groups strengthens the reliability of our findings.

The microbiological profile of diabetic foot infections varies considerably across geographical regions, and a substantial proportion of these infections are polymicrobial. In a 2021 systematic review and meta-analysis, McDonald et al. reported that *Staphylococcus aureus* was the most frequently isolated pathogen, followed by *Pseudomonas* spp., *Escherichia coli*, *Proteus* spp., *Klebsiella* spp., and *Enterococcus* spp. [8]. The authors further demonstrated that the distribution of Gram-positive and Gram-negative bacteria was associated with the Gross National Income of individual countries, suggesting that differences in healthcare delivery and sanitation may partly explain the observed regional variability. Similarly, in a systematic review and meta-analysis of diabetic foot infections from Sub-Saharan Africa, Wada et al. identified *Staphylococcus aureus* as the predominant pathogen, followed by *Escherichia coli* and *Pseudomonas aeruginosa* [23]. The authors emphasized that regional differences in bacterial distribution are likely influenced not only by geographical and environmental factors but also by infection duration, patient characteristics, and previous antibiotic exposure.

In the present study, 35.9% of infections were polymicrobial. The most frequently isolated microorganisms were *Staphylococcus* spp. (22.0%), *Escherichia coli* (14.8%), *Enterococcus* spp. (13.5%), and *Pseudomonas aeruginosa* (6.1%). Compared with reports from other geographical regions, our microbiological profile demonstrated both similarities and differences. As a tertiary regional referral center, our hospital receives a substantial number of patients with more complex diabetic foot infections referred from surrounding healthcare facilities. In addition, some patients may have received empirical antibiotic therapy before referral. These factors may have contributed to the microbiological profile observed in our cohort. However, owing to the retrospective design of the study, detailed information regarding prior antibiotic exposure, socioeconomic status, and other potential determinants was not available, precluding assessment of their relative contribution to the observed bacterial distribution.

Enterobacterales constitute one of the major Gram-negative pathogen groups implicated in diabetic foot infections. In our study, resistance to amoxicillin–clavulanate increased from 56.6% in the 2013–2016 period to 66.0% in the 2021–2024 period, while resistance to piperacillin–tazobactam increased from 12.5% to 38.3%; both increases were statistically significant. Notably, the nearly threefold increase in piperacillin–tazobactam resistance represents a clinically important finding. In a study conducted in Türkiye, Coşkun et al. (2024) reported resistance rates of 58.0%, 20.8%, and 52.1% to amoxicillin–clavulanate, piperacillin–tazobactam, and trimethoprim–sulfamethoxazole, respectively, among *Escherichia coli* isolates recovered from patients with diabetic foot infections [24]. Similarly, in a 2024 study conducted in Pakistan, Idrees et al. reported an amoxicillin–clavulanate resistance rate of 61.0% among *Escherichia coli* isolates recovered from patients with diabetic foot infections [25]. In a 2023 study conducted in Peru, Moya-Salazar et al. reported resistance rates of 36.0%, 14.0%, and 77.0% to amoxicillin–clavulanate, piperacillin–tazobactam, and trimethoprim–sulfamethoxazole, respectively, among *Escherichia coli* isolates recovered from patients with diabetic foot infections [26].

The increase in antimicrobial resistance observed among Enterobacterales cannot be attributed to a single factor. Wada et al. suggested that previous antibiotic exposure, duration of infection, and patient characteristics are among the factors that may influence bacterial distribution and antimicrobial resistance patterns [23]. Similarly, Moya-Salazar et al. reported that biofilm formation in chronic diabetic foot wounds may facilitate the development of antimicrobial resistance by exposing bacteria to prolonged courses of broad-spectrum antibiotics [26]. These findings suggest that the regional differences in antimicrobial resistance may result from the combined effects of multiple factors, including antibiotic prescribing practices, patient population characteristics, and the dynamics of chronic infections.

At our institution, amoxicillin–clavulanate and ciprofloxacin are among the most commonly prescribed oral antibiotics for the outpatient management of diabetic foot infections, whereas meropenem and piperacillin–tazobactam are frequently used in hospitalized patients with Gram-negative infections. Therefore, the increased resistance observed against these agents in our study may, at least in part, be associated with their widespread use in our clinical practice. However, owing to the retrospective design of our study, individual antibiotic exposure could not be evaluated, and thus this potential association could not be confirmed. These findings underscore the importance of institution-specific antimicrobial resistance surveillance to guide empirical antibiotic therapy and support the periodic revision of local treatment protocols.

The overall methicillin resistance rate among *Staphylococcus* spp. isolates was 62.2%. More notably, methicillin resistance reached 76% during the most recent study period, suggesting an increasing burden of methicillin-resistant *Staphylococcus* spp. in diabetic foot infections. No resistance to vancomycin or teicoplanin was detected throughout the study period. Although resistance rates increased over time for most of the antibiotics evaluated, a statistically significant increase was observed only for ciprofloxacin. In addition, increasing trends were noted for methicillin, levofloxacin, and fusidic acid resistance; however, these changes did not reach statistical significance.

In the 2024 study by Coşkun et al., resistance rates among *Staphylococcus* spp. isolates were reported as 64% for clindamycin, 58.1% for trimethoprim–sulfamethoxazole, and 40.5% for fusidic acid, while the methicillin resistance rate was 51.3%. Consistent with our findings, no resistance to vancomycin or teicoplanin was detected; however, trimethoprim–sulfamethoxazole resistance was higher than that observed in our cohort [24]. These findings suggest that considerable regional variations in antimicrobial resistance patterns may exist even within the same country. In a 2024 study conducted in China, Qi et al. reported a methicillin-resistant *Staphylococcus aureus* (MRSA) rate of 33.7% among *S. aureus* isolates and demonstrated that MRSA infections were associated with a higher prevalence of osteomyelitis, increased amputation rates, and prolonged hospital stays [27]. In contrast, an Australian study reported an MRSA rate of only 10%, despite a patient population in which 67% had moderate infections, 18% had severe infections, 46% had osteomyelitis, 58% had polymicrobial infections, and 64% had received antibiotics prior to hospital admission. Based on these findings, the authors concluded that routine empirical MRSA coverage might not be necessary in their center [28]. By comparison, a study from Poland reported an MRSA rate of 15.4%. Despite this relatively low prevalence, MRSA isolates exhibited high resistance rates to ciprofloxacin (93.5%), clindamycin (71%), and erythromycin (71%). In addition, 71% of the isolates were multidrug-resistant, whereas no resistance to vancomycin was detected [29]. Taken together, these findings indicate that the prevalence of MRSA in diabetic foot infections varies considerably not only between countries but also among tertiary referral centers with similar patient populations. Notably, despite the high prevalence of recognized risk factors for antimicrobial resistance in the Australian cohort, including severe infection, osteomyelitis, and prior antibiotic exposure, the reported MRSA rate remained remarkably low. This observation suggests that methicillin resistance cannot be explained solely by infection severity or patient characteristics and may also be influenced by local epidemiological factors and institutional antimicrobial resistance patterns. In this context, the increase in methicillin resistance to 76% during the most recent study period in our cohort indicates a substantial local burden of methicillin-resistant staphylococci and further emphasizes the importance of tailoring empirical antimicrobial therapy according to up-to-date local antimicrobial resistance surveillance data rather than relying solely on international estimates.

A major methodological strength of our study is that bacterial isolates were classified according to the clinically relevant antimicrobial susceptibility groups defined by the TMC-ADTS recommendations rather than by conventional taxonomic classification. The TMC-ADTS selective reporting system is based on the EUCAST susceptibility criteria and was developed to standardize reporting practices arising from the fact that routine clinical microbiology laboratories do not apply identical antimicrobial susceptibility panels to every bacterial species [14,30]. Because antimicrobial susceptibility panels vary according to the microorganism being tested, taxonomic classification may reduce the number of antibiotics that can be directly compared across different bacterial groups, thereby compromising the methodological robustness of temporal resistance analyses. Therefore, classifying bacterial isolates into clinically relevant groups with similar antimicrobial susceptibility panels enabled a more consistent and comparable evaluation of temporal resistance trends.

Another major methodological strength of our study is that temporal resistance analyses were conducted using predefined three-year study periods established on the basis of an a priori power analysis. This approach ensured adequate statistical power for evaluating temporal resistance trends in the Enterobacterales and *Staphylococcus* spp. groups. Furthermore, the 12-year study period enabled the assessment of long-term changes in antimicrobial resistance patterns rather than providing a cross-sectional snapshot of resistance at a single point in time. In addition, restricting the study population to patients who underwent toe amputation resulted in a more homogeneous cohort and reduced the potential clinical heterogeneity associated with different amputation levels. Finally, unlike many previously published cross-sectional studies, our study not only reports antimicrobial resistance rates during a specific period but also evaluates temporal changes in resistance patterns over a 12-year surveillance period.

Nevertheless, our study has several methodological limitations. First, because bacterial isolates were classified according to clinically relevant antimicrobial susceptibility groups, species-specific resistance patterns within the Enterobacterales group could not be analyzed separately. In addition, non-fermentative Gram-negative bacilli were evaluated as a single analytical group because species-level identification was unavailable for some isolates in the retrospective laboratory records. Consequently, resistance trends specific to *Pseudomonas aeruginosa*, *Acinetobacter* spp., and *Stenotrophomonas maltophilia* could not be assessed individually. Although adequate statistical power was achieved for temporal resistance analyses in the Enterobacterales and *Staphylococcus* spp. groups, the number of *Enterococcus* spp. and non-fermentative Gram-negative isolates was insufficient to perform reliable temporal analyses. Furthermore, owing to the retrospective design of the study, detailed information regarding diabetes duration, diabetic foot ulcer severity, and pre-admission antibiotic exposure, including the type, dosage, duration, and mode of antibiotic use, was unavailable. Finally, as this was a single-center study conducted at a tertiary referral hospital, the findings may not be directly generalizable to other healthcare settings or to the broader population of patients with diabetic foot infections.

This study provides comprehensive regional data on antimicrobial resistance patterns among bacterial isolates recovered from diabetic foot infections and underscores the importance of continuous microbiological surveillance and regular updating of empirical treatment protocols according to current local resistance data.

## 4. Materials and Methods

### 4.1. Study Design and Patient Selection

This retrospective, observational, and descriptive study was conducted at Baskent University, Adana Dr. Turgut Noyan Training and Research Center, a tertiary care medical center, with ethical approval obtained from the Baskent University Clinical Research Ethics Committee (Approval No. KA24/453). All demographic, clinical, microbiological, and antimicrobial susceptibility data were retrospectively extracted from the hospital’s digital healthcare information management system (Nucleus) independently by two investigators. Any discrepancies identified during data extraction were resolved by consensus. All data were anonymized prior to statistical analysis in accordance with the institutional ethics committee approval.

The study included 351 patients who underwent toe amputation due to diabetic foot infection between 2013 and 2024. Only patients who underwent toe amputation were included to obtain a relatively homogeneous study population. Patients who underwent foot debridement, transmetatarsal amputation, tarsal amputation, or more proximal lower-extremity amputations were excluded to minimize clinical heterogeneity. In addition, patients with a history of malignancy, rheumatologic disorders, traumatic amputation, or immunosuppressive therapy were excluded.

The variables collected included demographic characteristics, HbA1c levels, history of previous hospitalization, dialysis status, microorganisms isolated from wound cultures, and the corresponding antimicrobial susceptibility profiles.

### 4.2. Culture Collection and Microbiological Evaluation

Culture specimens were obtained using two sampling methods. Samples were collected either from intraoperative tissue specimens obtained from infected or necrotic tissue or from deep wound swabs collected from the central area of the ulcer using a sterile applicator. All specimens were obtained under aseptic conditions and processed according to standard microbiological procedures.

Clinical specimens were inoculated onto blood agar (cat. no. 1108.00) and MacConkey agar (cat. no. 1052.00; both from Condalab, Madrid, Spain) and incubated aerobically at 35 ± 2 °C for 18–24 h. For bacterial identification and antimicrobial susceptibility testing (AST), bacterial suspensions were prepared and adjusted to a 0.5 McFarland turbidity standard (acceptable range: 0.4–0.6). Bacterial identification was performed using the VITEK 2 Compact system (bioMérieux, Marcy-l’Étoile, France). Antimicrobial susceptibility testing was performed using the VITEK 2 Compact system and supplemented by the Kirby–Bauer disk diffusion method on Mueller–Hinton Agar II (Condalab, cat. no. 1055.00). Antibiotic disks (Bioanalyse, Ankara, Türkiye) and gradient diffusion strips (Etest, bioMérieux) were applied to the inoculated agar surfaces. The plates were incubated at 35 ± 1 °C for 18 ± 2 h in ambient air or, when required, in an atmosphere supplemented with 5% CO_2_. Zone diameters and minimum inhibitory concentrations (MICs) were interpreted according to the clinical breakpoints and expert rules of the European Committee on Antimicrobial Susceptibility Testing (EUCAST) [30]. The antimicrobial susceptibility results were subsequently categorized according to the 2022 Turkish Microbiology Society Antibiotic Susceptibility Testing Standards (TMC ASTS) Short Notification Form [14].

### 4.3. Bacterial Classification and Grouping by Time Periods

The isolated microorganisms were classified into five major microbiological groups according to the 2022 Turkish Society for Microbiology Antibiotic Susceptibility Testing Standards (TMC ASTS) Short Notification Form [14], which categorizes microorganisms according to similar antimicrobial susceptibility reporting algorithms. The predefined microbiological groups used for analysis were as follows:

Enterobacterales;
*Staphylococcus* spp.;
*Streptococcus* spp.;
*Enterococcus* spp.
Non-fermentative Gram-negative bacilli

The non-fermentative Gram-negative bacilli category was included as a study-specific analytical group because species-level identification was not consistently available for all isolates in the retrospective laboratory records. This category comprised isolates reported as non-fermentative Gram-negative bacilli, as well as identified non-fermenting organisms such as *Pseudomonas aeruginosa*, *Acinetobacter* spp., and *Stenotrophomonas maltophilia*.

To ensure an adequate number of culture-positive isolates for temporal comparisons and binary logistic regression analysis, the study period was divided into three predefined intervals. Each interval contained at least 25 culture-positive isolates. A post hoc power analysis was performed using G*Power version 3.1. Assuming resistance rates between 20% and 60% and a significance level of 5%, the estimated statistical power of the study was approximately 90%. Accordingly, the microbiological data were analyzed across three consecutive time periods:

Group 1: 2013–2016;
Group 2: 2017–2020;
Group 3: 2021–2024.

The microorganisms isolated during each study period and their corresponding antimicrobial susceptibility profiles were retrospectively compared.

### 4.4. Statistical Analysis

This study utilized IBM SPSS Statistics, version 26, for statistical analysis. The distribution of numerical variables was evaluated via visual inspection (including histograms and skewness–kurtosis plots) and the Kolmogorov–Smirnov test. The associations between categorical variables and group classifications were analyzed utilizing the Chi-square test or Fisher’s exact test, contingent upon the appropriateness of the data. Temporal variations in antibiotic resistance were examined using a binary logistic regression model. The study period was entered into the logistic regression model as an ordinal variable (1 = 2013–2016, 2 = 2017–2020, and 3 = 2021–2024). A *p*-value of less than 0.05 was considered statistically significant for all analyses.

## Figures and Tables

**Table 1:** Demographic and clinical characteristics of the patients included in the study.

Variable	*n* (%)	Mean ± SD (Min–Max)	*p*-Value
**Total number of patients**	351		
**Gender**			
**–Male**	225 (64%)		
**–Female**	126 (36%)		
**Age (years)**		64.1 ± 10.7 (32–94)	
**–Male**		63.4 ± 11.1	0.178
**–Female**		65.0 ± 10.3	
**Chronic kidney disease**	207 (58.9%)		
**Patients receiving dialysis treatment**	105 (30%)		
**Patients with history of hospitalization**	190 (54%)		
**HbA1c**		8.9 ± 2.0 (5.44–15)	

SD: Standard deviation; HbA1c: Hemoglobin A1c. The difference in mean age between the two groups was assessed using an independent samples *t*-test.

**Table 2:** Distribution of microbial isolates recovered from positive cultures.

Microbiological Group	Species Included	*n*	%
***Staphylococcus* spp. (*n* = 84, 22.0%)**	Coagulase-negative *Staphylococcus*	50	13.2
	*Staphylococcus aureus*	34	9.0
***Streptococcus* spp. (*n* = 25, 6.6%)**	Group D *Streptococcus*	6	1.6
	Group G β-hemolytic *Streptococcus*	1	0.3
	*Streptococcus agalactiae*	3	0.8
	*Streptococcus mutans*	1	0.3
	*Streptococcus* sp.	14	3.4
***Enterococcus* spp. (*n* = 51, 13.5%)**	*Enterococcus* spp.	51	13.5
**Non-fermentative Gram-negative bacilli (*n* = 39, 10.6%)**	*Pseudomonas aeruginosa*	23	6.1
	Non-fermentative Gram-negative bacilli	9	2.4
	*Acinetobacter baumannii*	5	1.3
	*Acinetobacter lwoffii*	1	0.3
	*Stenotrophomonas maltophilia*	1	0.3
**Enterobacterales (*n* = 167, 44.2%)**	*Escherichia coli*	56	14.8
	*Morganella morganii*	24	6.3
	*Klebsiella pneumoniae*	17	4.5
	*Proteus mirabilis*	16	4.2
	*Enterobacter aerogenes*	11	2.9
	*Serratia marcescens*	11	2.9
	*Proteus vulgaris*	10	2.6
	*Enterobacter cloacae*	6	1.6
	*Citrobacter freundii*	4	1.1
	*Klebsiella oxytoca*	4	1.1
	*Proteus penneri*	3	0.8
	*Providencia rettgeri*	2	0.5
	*Serratia liquefaciens*	2	0.5
	*Pantoea agglomerans*	1	0.3
**Others (*n* = 12, 3.2%)**	Diphtheroid bacillus	5	1.3
	*Candida albicans*	4	1.1
	*Bacillus capillosus*	1	0.3
	*Fusobacterium varium*	1	0.3
	*Micrococcus* sp.	1	0.3

The table presents the distribution of microorganisms isolated from positive cultures. Isolates were classified into five major microbiological groups according to the Turkish Society of Microbiology Antimicrobial Susceptibility Testing Standards short notification form [14].

**Table 3:** Distribution of bacterial groups according to gender, dialysis status, and hospitalization history.

Bacterial Group	F (%)	M (%)	D+ (%)	D− (%)	H+ (%)	H− (%)
***Staphylococcus* spp.**	20.8	22.6	21.3	22.8	23.9	28.2
***Streptococcus* spp.**	8.3	5.6	5.6	8.7	9.8	7.7
***Enterococcus* spp.**	14.6	12.8	10.2	16.0	13.0	17.9
**Enterobacterales**	46.5	42.7	52.8	37.9	44.6	38.5
**Non-fermentative Gram-negative bacilli**	6.9	12.8	7.4	11.2	3.3	5.1
**Others**	2.8	3.4	2.8	3.4	2.6	5.4

Values are presented as percentages. Abbreviations: F, female; M, male; D+, dialysis; D−, no dialysis; H+, previous hospitalization; H−, no previous hospitalization. Comparisons were performed using the chi-square test (gender, *p* = 0.454; dialysis, *p* = 0.186; previous hospitalization, *p* = 0.738).

**Table 4:** Antibiotic resistance rates of Enterobacterales across the three consecutive time periods.

Antibiotic	Total Resistance (%)	2013–2016 (%)	2017–2020 (%)	2021–2024 (%)	*p* Value	Exp(B)	95% CI for Exp(B) (Lower–Upper)
**Ampicillin** (10 µg)	91.1	89.1	93.6	91.5	0.616	1.188	0.606–2.327
**Amoxicillin–clavulanate** * (20–10 µg)	51.6	40.6	52.1	66.0	0.009 *	1.677	1.137–2.474
**Ampicillin–sulbactam** (10–10 µg)	37.7	34.4	33.3	46.8	0.207	1.280	0.871–1.894
**Piperacillin–tazobactam** * (30–6 µg)	17.0	12.5	2.1	38.3	0.002 *	2.399	1.389–4.146
**Cefuroxime sodium** (30 µg)	57.9	54.7	47.9	68.1	0.186	1.296	0.882–1.904
**Cefotaxime** (5 µg)	30.8	29.7	27.1	36.2	0.502	1.149	0.766–1.724
**Ceftriaxone** (30 µg)	30.2	28.1	25.0	38.3	0.286	1.249	0.830–1.880
**Gentamicin** (10 µg)	21.4	15.6	25.0	25.5	0.191	1.358	0.859–2.147
**Amikacin** (30 µg)	4.4	3.1	6.3	4.3	0.728	1.175	0.474–2.917
**Trimethoprim–sulfamethoxazole** * (1.25–23.75 µg)	30.8	21.9	29.2	44.7	0.012 *	1.701	1.122–2.579
**Ciprofloxacin** (5 µg)	41.5	39.1	37.5	48.9	0.327	1.210	0.826–1.772
**Levofloxacin** (5 µg)	40.9	39.1	35.4	48.9	0.336	1.206	0.823–1.768
**Imipenem** ** (10 µg)	5.7	1.6	6.3	10.6	0.054 **	2.463	0.986–6.152
**Meropenem** * (10 µg)	6.3	1.6	6.3	12.8	0.027 *	2.779	1.124–6.872

Antibiotic resistance trends were analyzed using binary logistic regression. Exp(B) represents the odds ratio, and CI indicates the 95% confidence interval. The values in parentheses following each antibiotic represent the antibiotic disk content (µg) used for antimicrobial susceptibility testing. A single asterisk (*) indicates statistical significance (*p* < 0.05), whereas a double asterisk (**) indicates borderline statistical significance (0.05 ≤ *p* < 0.10).

**Table 5:** Antibiotic resistance of *Staphylococcus* spp. across the three consecutive time periods.

Antibiotic	Total Resistance (%)	2013–2016 (%)	2017–2020 (%)	2021–2024 (%)	*p* Value	Exp(B)	95% CI for Exp(B) (Lower–Upper)
**Penicillin (1 µg)**	92.7	92.0	96.9	88.0	0.589	0.742	0.252–2.190
**Methicillin (Oxacillin (1 µg)**	62.2	52.0	59.4	76.0	0.084	1.686	0.933–3.047
**Erythromycin (15 µg)**	58.5	48.0	59.4	68.0	0.154	1.519	0.855–2.700
**Clindamycin (2 µg)**	39.0	28.0	50.0	64.0	0.563	1.184	0.669–2.093
**Vancomycin (Etest)**	0	0	0	0	—	—	—
**Teicoplanin (Etest)**	0	0	0	0	—	—	—
**Fusidic acid (10 µg)**	31.7	24.0	28.1	56.0	0.132	1.602	0.867–2.958
**Trimethoprim–sulfamethoxazole (1.25–23.75 µg)**	17.1	12.0	18.8	20.0	0.454	1.220	0.630–2.804
Ciprofloxacin * (5 µg)	40.2	20.0	50.0	48.0	0.047 *	1.822	1.009–3.292
**Levofloxacin (5 µg)**	39.0	20.0	50.0	44.0	0.085	1.675	0.931–3.015

Antibiotic resistance trends were analyzed using binary logistic regression. The values in parentheses following each antibiotic represent the antibiotic disk content (µg) used for antimicrobial susceptibility testing. Exp(B) indicates the odds ratio, and CI represents the confidence interval. A single asterisk (*) denotes statistical significance (*p* < 0.05).

**Table 6:** Antibiotic resistance of *Enterococcus* spp., *Streptococcus* spp., and Gram-negative non-fermentative bacilli.

Antibiotic (*Enterococcus* spp.)	Resistance (%)	Antibiotic (*Streptococcus* spp.)	Resistance (%)	Antibiotic (Gram-Negative Non-Fermentative Bacilli)	Resistance (%)
Penicillin (2 µg)	11.8	Penicillin (1 µg)	20.0	Piperacillin–tazobactam (30–6 µg)	24.3
Vancomycin (5 µg)	5.9	Clindamycin (2 µg)	12.0	Amikacin	21.6
Teicoplanin (30 µg)	5.9	Vancomycin (5 µg)	5.0	Ceftazidime (10 µg)	24.3
Streptomycin (300 µg)	27.5	Teicoplanin (30 µg)	5.0	Ciprofloxacin (5 µg)	37.8
Ampicillin (2 µg)	11.8	Ampicillin (2 µg)	20.0	Levofloxacin (5 µg)	27.0
Gentamicin (30 µg)	23.5	Ceftriaxone (30 µg)	28.0	Imipenem (10 µg)	24.3
—	—	—	—	Meropenem (10 µg)	24.3
—	—	—	—	Cefepime (30 µg)	21.6

Resistance percentages represent overall resistance rates The values in parentheses following each antibiotic represent the antibiotic disk content (µg) used for antimicrobial susceptibility testing.

## Data Availability

The data presented in this study are available from the corresponding author upon reasonable request.
